# Risk Factors for Excess Mortality in the First Year After Curative Surgery for Colorectal Cancer

**DOI:** 10.1245/s10434-012-2294-6

**Published:** 2012-03-07

**Authors:** Gea A. Gooiker, Jan Willem T. Dekker, Esther Bastiaannet, Lydia G. M. van der Geest, Jos W. S. Merkus, Cornelis J. H. van de Velde, Rob A. E. M. Tollenaar, Gerrit-Jan Liefers

**Affiliations:** 1 Department of Surgery, Leiden University Medical Centre, Leiden, The Netherlands; 2 Comprehensive Cancer Centre, Leiden, The Netherlands; 3 Department of Surgery, HAGA Hospital, The Hague, The Netherlands

## Abstract

**Background:**

Thirty-day mortality after surgery for colorectal cancer may vastly underestimate 1-year mortality. This study aimed to quantify the excess mortality in the first postoperative year of stage I–III colorectal cancer patients and to identify risk factors for excess mortality.

**Methods:**

All 2,131 patients who were operated with curative intent for stage I–III colorectal cancer in the western region of the Netherlands between January 1, 2006, and December 31, 2008, were analyzed. Thirty-day mortality and relative survival were calculated. In addition, relative excess risk (RER) of death was estimated by a multivariable model.

**Results:**

Thirty-day mortality was 4.9%. One-year mortality was 12.4%. Risk factors for excess mortality in the first postoperative year for colon cancer patients were emergency surgery (excess mortality 29.7%, RER 2.5, 95% confidence interval 2.5–5.0), a Charlson score of >1 (excess mortality 12.6%, RER 2.3, 95% confidence interval 1.5–3.7), stage II or III disease (excess mortality 14.9%, RER 3.9, 95% confidence interval 1.9–8.1), and postoperative adverse events (excess mortality 22.6%, RER 2.1, 95% confidence interval 1.4–3.2).

**Conclusions:**

The 30-day mortality rate highly underestimates the risk of dying in the first year after surgery, with excess 1-year mortality rates varying from 15 to 30%. This excess mortality was especially prominent in patients with comorbidities, higher stages of disease, emergency surgery, and postoperative surgical complications.

**Electronic supplementary material:**

The online version of this article (doi:10.1245/s10434-012-2294-6) contains supplementary material, which is available to authorized users.

Adverse postoperative events are most often recorded as 30-day mortality and postoperative complications. Large studies show that colorectal cancer surgery can be considered as high risk, with reported postoperative mortality and complication rates of approximately 5% and 20–40%, respectively.^1^–^4^

For the prognosis of colorectal cancer patients who undergo curative surgery, adverse postoperative events are a determining factor. Several studies showed that complications are of prognostic importance because they can cause delayed mortality.^5^,^6^ Furthermore, specific complications, such as anastomotic leaks, are associated with local recurrences and reduced survival.^7^–^9^ Elderly patients with severe comorbidities, lower socioeconomic status, stage III tumor, or preoperative tumor complications have been demonstrated to be at higher risk for postoperative adverse events.^10^–^12^

Earlier studies have shown that 30-day mortality after surgery for colorectal cancer vastly underestimates 1-year mortality, even in young colorectal cancer patients who were operated with curative intent.^13^,^14^ Furthermore, Dekker et al. showed that 1-year excess mortality (1-year mortality adjusted for expected mortality in the general population) was the main determining factor for age-related survival differences: after surviving the first year, elderly patients had the same cancer-related survival as younger patients.^14^ This suggests that there may be a prolonged effect of the assault of surgery. To date, there is little information on the etiology of the excess mortality in the first postoperative year. Because of the lack of reported determinants of excess mortality in the contemporary literature, we set out to determine the role of comorbidity, emergency surgery, and postoperative complications, expecting that these factors are main determinants for 1-year excess mortality, especially in elderly patients, where the incidence of comorbidity is higher and emergency surgery and postoperative complications occur more frequently. The identification of risk factors would help to stratify patients according to their risk. Moreover, risk factors may be modifiable and targeted optimization of care may result in an improved prognosis.

Therefore, the first aim of this study was to quantify the excess mortality in the first postoperative year of stage I–III colorectal cancer patients operated with curative intent according to several patient and tumor characteristics. The second aim of this study was to identify risk factors for excess mortality in the first postoperative year.

## Methods

### Data

In the Netherlands, all newly diagnosed malignancies are registered in the nationwide population based Netherlands Cancer Registry. The Leiden Cancer Registry, part of the Netherlands Cancer Registry, collects data on all cancer patients diagnosed in one of the nine affiliated hospitals in the midwestern part of the Netherlands. This region comprises one university hospital, six teaching hospitals, and two nonteaching hospitals, and serves a population of 1.7 million inhabitants.

Independently trained data managers collected data from the original patient files after receiving an automatic report from the Dutch pathology reporting system, PALGA. Information on patient characteristics, tumor characteristics, treatment, hospital of diagnosis, and/or treatment and follow-up were recorded. Tumor site and morphology were coded according to the International Classification of Diseases for Oncology.^15^ Cancers were staged according to the tumor, node, metastasis system classification for malignant tumors, 6th edition.^16^ The quality of the data was high, and completeness was estimated to be at least 95%.^17^,^18^

In 2006, a regional audit for colorectal cancer surgery (KIC) was started in the nine affiliated hospitals of the Leiden Cancer Registry. The data collection was extended to data that reflected quality of care and case-mix factors. These prospectively collected data were used for benchmarking and feedback on each hospital’s process, ultimately to improve the quality of care in the entire region. Data were collected on patient demographics (age, sex, comorbidities, socioeconomic status), tumor characteristics (localization, tumor, node, metastasis system staging), and treatment characteristics (neoadjuvant or adjuvant treatment, type of surgery, emergency, hospital of treatment, surgical complications, length of stay). Apart from American Society of Anesthesiology (ASA) scores, completeness of data was more than 98%.

### Patients

All patients who had a resection of stage I, II, or III tumor of the colon (C18), the rectosigmoid (C19), or the rectum (C20) from January 1, 2006, to December 31, 2008, in one of the affiliated hospitals were identified. Excluded were patients with stage IV tumor, patients with a tumor of the appendix, and patients who did not undergo tumor resection. Age was categorized as younger than 65 years, 65–74 years, and 75 years and older. Comorbidity was recorded according to a slightly modified version of the Charlson index used by the Dutch Cancer Registry. ASA scores were categorized as ASA I and II, or ASA III and IV. Missing data from the ASA score were included as a separate category. Socioeconomic status was categorized as low, intermediate, and high on the basis of area-based data concerning income, employment, and education provided by the Netherlands Institute for Social Research.^19^ Surgical complications comprised superficial wound infections, abdominal wall problems (i.e., dehiscence), deep infections, and intra-abdominal complications, including bleeding, ileus, abscess, or anastomotic leaks. As a substitute for overall complications (both surgical and nonsurgical, such as pulmonary or cardiac events), a prolonged length of stay was used, which was defined as a hospital admission of 15 days or longer after surgery. Vital status of all patients was obtained actively on a regular basis through linkage of the cancer registry data with the integrated database of the municipal registry and the central bureau for genealogy. Follow-up was completed until January 1, 2010.

### Statistical Analysis

All analyses were performed for colon and rectum cancer patients separately. Stratified by several characteristics, 30-day mortality, 1-year overall mortality (all causes), and 1-year excess mortality rates were calculated. Excess mortality was calculated by the following formula: [(Observed number of deaths in the first year − Expected number of deaths in the matched general population)/Number of patients]. The expected number of deaths was calculated by national life tables matched for age, sex, and year of incidence. Survival curves of the relative survival in the first year were constructed. Relative survival is the preferred way to describe the prognosis of elderly cancer patients because it takes into account the risk of dying of other causes than the disease of interest in the absence of cause of death in the database. Relative survival was calculated as the ratio of observed survival among the cancer patients to expected survival. Relative excess risk (RER) of death and *P* values were estimated by a multivariable generalized linear model with a Poisson distribution based on collapsed relative survival data based on exact survival times.

## Results

From January 1, 2006, to December 31, 2008, a total of 2131 patients had a colorectal resection in one of the nine affiliated hospitals for stage I–III colorectal cancer; 1407 underwent treatment for colon cancer and 724 for rectal cancer. Table 1 lists the characteristics of the study population for colon and rectal cancer patients. Colon patients were older, more often female, had more comorbidity, and more often required emergency surgery.

**Table 1:** Characteristics of the population according to localization

Characteristic	Colon cancer (*n* = 1407)	Rectal cancer (*n* = 724)
Age (years)		
<65	371 (26.4%)	264 (36.5%)
65–74	390 (27.7%)	236 (32.6%)
≥75	646 (45.9%)	244 (30.9%)
Sex		
Male	672 (47.8%)	406 (56.1%)
Female	735 (52.2%)	318 (43.9%)
Stage		
I	234 (16.6%)	214 (29.6%)
II	661 (47.0%)	231 (31.9%)
III	512 (36.4%)	279 (38.5%)
Emergency		
Emergent	188 (13.4%)	17 (2.4%)
Elective	1219 (86.6%)	707 (97.6%)
ASA		
I/II	633 (45.0%)	411 (56.8%)
III/IV/V	297 (21.1%)	123 (17.0%)
Unknown	477 (33.9%)	190 (26.2%)
Charlson		
0	757 (53.8%)	444 (61.3%)
1	358 (25.4%)	160 (22.1%)
2 or more	292 (20.8%)	120 (16.6%)
Complications		
No	1155 (82.1%)	559 (77.2%)
Yes	252 (17.9%)	165 (22.8%)
Hospital stay		
<15 days	956 (67.9%)	471 (65.0%)
≥15 days	419 (29.8%)	238 (32.9%)
Unknown	32 (2.3%)	15 (2.1%)
Socioeconomic status		
High	449 (31.9%)	243 (33.6%)
Intermediate	480 (34.1%)	241 (33.3%)
Low	478 (34.0%)	240 (33.1%)

*ASA* American Society of Anesthesiology

### Mortality

**Fig. 1:**
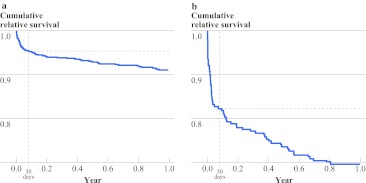
Relative survival in the first year. **a** All patients. **b** Colon cancer patients treated in an emergency setting. The *horizontal dotted line* marks day 30 day after surgery. The *blue line* indicates the relative survival in the first year. The *space* between the *vertical dotted line* and the *blue line* represents the excess mortality after 30 days and within the first year after surgery

The overall observed 30-day mortality was 4.9%, and 1-year mortality was 12.4%. Median follow-up time was 24.6 (range 0.03–47.9) months. All patients had follow-up for at least 1 year unless they died before then. Table 2 shows crude overall mortality and excess mortality rates of stage I–III colon and rectal cancer patients in the first year after surgery according to several characteristics. The observed 1-year mortality rates of patients older than 75 were 21 and 16% for colon and rectal patients, respectively. High excess mortality rates were observed in colon cancer patients with ASA III/IV (19.9%), a stage III tumor (14.9%), and a Charlson score of 2 or higher (17.5%), and in patients with postoperative surgical complications (22.6%) or a prolonged length of stay (22%). Of all colon cancer patients with an emergency resection, 33% died in the first year, an excess mortality of 30%. In rectal cancer patients, high excess mortality rates were observed in elderly patients (8.9%), patients with a Charlson score of 2 or higher (13%), and patients with postoperative complications (11.2%). Figure 1 shows relative survival curves of all patients in the cohort (Fig. 1a) and an example of relative survival in a specific subgroup: colon cancer patients treated in an emergency setting (Fig. 1b).

**Table 2:** Overall 30-day and 1-year mortality, and 1-year excess mortality

Characteristic	Colon			Rectal		
	30-days mortality	1-year overall mortality	1-year excess mortality	30-days mortality	1-year overall mortality	1-year excess mortality
Overall	6.3	14.8	10.9	2.4	7.9	4.8
Sex						
Male	7.7*	16.2	12.1	3.5*	9.6	6.2
Female	4.9*	13.5	9.8	0.9*	5.7	3.2
Age						
<65	1.6*	5.9*	5.3*	0.4*	2.3*	1.6*
65–74	4.1*	12.3*	10.2*	0.4*	6.8*	4.5*
≥75	10.2*	21.4*	14.6*	6.7*	15.6*	8.9*
Socioeconomic status						
High	4.7*	12.9	9.0	2.5	7.0	3.5
Intermediate	8.5*	15.2	11.5	2.9	9.1	6.7
Low	5.4*	16.1	12.1	1.7	7.5	4.2
ASA						
I/II	1.9*	7.9*	4.4*	1.0*	5.1*	2.3*
III/IV	11.1*	24.9*	19.9*	6.5*	14.6*	10.2*
Unknown	9.0*	17.6*	13.8*	2.6*	9.5*	6.6*
Stage						
I	6.8	9.8*	6.1*	1.9	4.7	1.3
II	5.9	13.8*	9.5*	3.0	8.2	5.4
III	6.5	18.4*	14.9*	2.2	10.0	7.1
Emergency						
Emergent	18.1*	32.9*	29.7*	NA		
Elective	4.4*	11.9*	8.0*			
Comorbidity						
No	4.3*	10.5*	7.7*	0.3*	4.4*	1.7*
Yes	7.7*	17.8*	13.2*	4.5*	11.5*	8.0*
Charlson						
0	4.1*	10.8*	7.5*	0.7*	4.7*	1.8*
1	6.7*	17.0*	12.6*	3.8*	10.0*	6.9*
2 or more	11.3*	22.3*	17.5*	6.7*	16.7*	13.0*
Complications						
No	5.2*	12.4*	8.3*	1.4*	6.1*	2.9*
Yes	11.1*	25.8*	22.6*	5.5*	13.9*	11.2*
Hospital stay						
<15 days	5.4	9.8*	6.2*	2.3	5.5*	3.0*
≥15 days	8.4	26.5*	22.0*	2.5	11.3*	7.2*
Unknown	3.1	9.4*	5.0*	0	26.7*	24.3*

*ASA* American Society of Anesthesiology

* *P* < 0.05 for the different value within a variable

### Risk Factors for Excess Mortality in the First Year

**Fig. 2:**
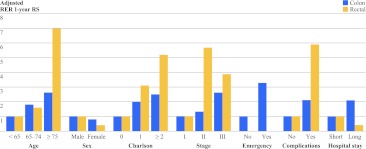
Factors associated with 1-year relative survival in multivariable analysis for colon and rectal cancer patients. At the *y* axis, the effect size is represented as adjusted relative excess risk (RER) for 1-year mortality, compared to the reference group, which always has a RER of 1

Figure 2 shows all identified risk factors for 1-year mortality for colon and rectal cancer patients separately. The effect is represented as adjusted RER for 1-year mortality. In Supplementary Table, the results of univariate and multivariable analyses for risk factors for excess mortality in the first year are shown.

For colon cancer patients, significant risk factors for excess mortality in the first year after surgery were a stage III tumor [RER 2.6, 95% confidence interval (CI) 1.3–5.3, *P* < 0.001], a Charlson score of >1 (RER 2.5, 95% CI 1.6–4.0, *P* = 0.001), an emergency resection (RER 3.3, 95% CI 2.5–5.), *P* < 0.001), and postoperative surgical complications (RER 2.1, 95% CI 1.4–3.3, *P* < 0.001). Particular comorbidities that increase the risk of excess mortality were a previous tumor (RER 1.8, 95% CI 1.2–2.8, *P* = 0.009), pulmonary disease (RER 2.1, 95% CI 1.3–3.2, *P* = 0.001), gastrointestinal disease (RER 2.0, 95% CI 1.1–3.6, *P* = 0.02), and neurologic disease (RER 1.7, 95% CI 1.0–2.8, *P* = 0.04).

For rectal cancer patients risk factors for excess mortality in the first year, were age ≥75 years (RER 7.0, 95% CI 1.8–27.4, *P* = 0.009), Charlson score of >1 (RER 5.2, 95% CI 1.7–15.9, *P* = 0.01), and postoperative surgical complications (RER 5.9, 95% CI 1.3–26.8, *P* = 0.02). Particular comorbidities that increased the risk were hypertension (RER 2.4, 95% CI 1.1–5.5, *P* = 0.04), vascular disease (RER 3.4, 95% CI 1.3–9.3, *P* = 0.02), kidney disease (RER 7.9, 95% CI 2.4–26.5, *P* = 0.001), and neurologic disease (RER 3.3, 95% CI 1.0–10.4, *P* = 0.04).

## Discussion

The excess mortality in the first year after surgery for stage I–III colorectal cancer is high. Overall, 12.4% of all patients died within the first year, compared to a 4.9% 30-day mortality rate. Thus, the 30-day mortality rate greatly underestimates the risk of dying in the first year after surgery. After adjustment for expected mortality in the general population, patients with comorbidities, patients with stage III tumors, patients requiring emergency resection, and patients with postoperative surgical complications were at higher risk for excess mortality, with excess 1-year mortality rates varying from 15 to 30%.

To our knowledge, the present study is the first detailed population-based study to quantify excess mortality after colorectal cancer surgery with curative intent and to examine risk factors for excess mortality within the first year. Clinical, accurate data of the Netherlands Cancer Registry were used, and variables not only comprised age and stage, but also data on comorbidities and emergency and postoperative complications. Moreover, relative survival and excess mortality rates were used as outcome measures. This also takes into account mortality not attributable to the examined disease or the treatment of the disease.

The results in this study should be interpreted with regard to several limitations inherent to its observational design. In the univariate and multivariable analyses, potential confounders were examined and added to the model (Supplementary Table). In calculations of expected mortality, patients with severe comorbidity may not match well with the general population. This could have led to a slight underestimation of the expected mortality in this group, resulting in a lower excess mortality.

Furthermore, only data on postoperative surgical complications were available. To estimate the impact of both surgical and nonsurgical complications (e.g., pneumonia, delirium, cardiac event, urinary tract infection), a prolonged length of stay was used as a substitute of postoperative complications in general. In this study, a prolonged length of stay was defined as a stay of 15 days or longer. An uneventful postoperative period is unlikely to result in a longer hospital stay, and therefore, it can function as a proxy for overall complications. This assumption is in line with a study of Cohen et al., who demonstrated a mean length of stay after colorectal surgery of 16 days in the presence of complications, versus 6 days when no complications occurred.^20^ In the present study, a prolonged length of stay occurred in 30.4% of the patients. This is also consistent with findings in the literature.^20^,^21^

Despite these limitations, the present study provides valuable information, showing that 30-day mortality underreports postoperative mortality after colorectal surgery. This is consistent with previous studies.^13^,^14^ Visser et al. reported a doubling of 30-day mortality to 9.1% at 90 days after surgery.^13^ A previous study from our group showed that 1-year excess mortality was the main determinant for age-related survival differences.^14^ In the present data, the steepest decline in relative survival was observed during the first 7–11 months after surgery. The excess mortality was especially high in patients with comorbidities, stage III disease, emergency surgery, postoperative surgical complications, and a prolonged length of stay. These risk factors have been previously described as risk factors for postoperative mortality and survival.^11^,^12^,^22^,^23^ However, these reports did not adjust for expected mortality in the general population, thereby not taking into account the risk of dying of other causes than colorectal cancer.

The aim of identifying risk factors for 1-year excess mortality was to find targets for improvement of patient care. However, these risk factors may not be easily malleable.

### Comorbidity

The prevalence of comorbid disease is increasing with the aging population and improvements in modern medicine.^24^,^25^ Patients with comorbidity may have less biologic reserve, and comorbidities alter organ functions.^26^ More research is needed on these mechanisms and the influence of comorbidities on postoperative outcomes. Obviously, optimizing perioperative care to reduce surgical risk by thorough preoperative assessment and by additional supportive measures may improve the prognosis of patients. A multidisciplinary approach with integrated chronic disease management in cancer patients in the posthospital period also seems warranted.

### Stage III Disease

Patients with stage III disease had an increased risk of excess 1-year mortality. This could be due to cancer recurrences, although it is unlikely a large part of early mortality is due to cancer recurrences in patients operated with curative intent. An earlier study on relapses in these patients showed that only a small number of patients experienced a recurrence within the first year. Furthermore, only 10% of patients with a recurrence die within 1 year.^27^

### Emergency Surgery

Emergency surgery has consistently been demonstrated to be a major risk factor for adverse outcome in colorectal surgery. Efforts should be made to reduce the number of patients in need of an emergent intervention. In this respect, national screening programs could be helpful. If colorectal cancer could be identified at an earlier (asymptomatic) stage, it could be expected that the need for emergent surgery may be reduced.

### Postoperative Surgical Complications or a Prolonged Length of Stay

Patients with postoperative surgical complications or a prolonged length of stay had an increased risk for excess mortality in the first year. These results compare to a recent study by Greenblatt et al.^5^ They showed that readmission after colectomy due to a postoperative complication was predictive for 1-year mortality. Two studies by Ghaferi et al. showed that differences in death after major complications were the primary determinant of variation of mortality between hospitals.^28^,^29^ This indicates that effective management of postoperative complications may reduce postoperative mortality and improve patient outcomes. This provides a potential target for improvement of the quality of cancer care. Not only prevention of complications but also early recognition and aggressive treatment of complications can improve patient outcomes. Identifying structures, processes, and best practices to reduce the occurrence of complications and improve the management of complications should have priority. Although randomized, controlled trials are crucial for determination of efficacious interventions, large cohort studies and comparative effectiveness research are essential to fill critical gaps in defining optimal strategies for complication management.^30^

## Conclusions

In conclusion, the excess mortality in the first postoperative year after colorectal cancer surgery is high and reflects postoperative risk more accurately than 30-day mortality. The presence of comorbidities, stage III disease, emergency resection, and postoperative surgical complications were predictive for excess mortality, with excess 1-year mortality rates as high as 15–30%. These risk factors may not be easily modifiable. Nevertheless, their identification is important to develop tailored treatment of high-risk patients. Moreover, identifying effective strategies for both prevention and treatment of complications could have the potential to improve patient outcomes.

## Electronic Supplementary Material

Below is the link to the electronic supplementary material.

Supplementary material 1 (PDF 40.7 kb)
