# Supplementary material for: Risk Factors for Excess Mortality in the First Year After Curative Surgery for Colorectal Cancer
Source: Ann Surg Oncol. 2012 Mar 7;19(8):2428–34. doi: 10.1245/s10434-012-2294-6 (PMC3404283; doi:10.1245/s10434-012-2294-6)
Supplement: Supplementary file 1 — Supplementary material 1 (PDF 40.7 kb) [file 10434_2012_2294_MOESM1_ESM.pdf]

Supplementary table to figure 2: Multivariable One-year Relative Survival, expressed as relative excess risk (RER) for colon and rectal cancer patients separately Multivariable analyses includes all variables with p-value of 0.1 or smaller. Besides, gender, stage & treatment were also entered into the model.

		EVENT = DOD AT 1 YEAR							
		COLON				RECTAL			
		Univariate RER (95%CI)	p-value	Multivariable RER (95%CI)	p-value	Univariate RER (95%CI)	p-value	Multivariable RER (95%CI)	p-value
**Age**	<65	***1***	***0.0001***	***1***	***0.004***	***1***	***0.01***	***1***	***0.009***
	65-74	***2.0 (1.1-3.6)***		***1.8 (1.0-3.3)***		***2.8 (0.7-11.0)***		***1.6 (0.4-7.6)***	
	≥75	***3.2 (1.9-5.4)***		***2.6 (1.5-4.5)***		***6.2 (1.7-22.0)***		***7.0 (1.8-27.4)***	
**Sex**	Male	1	0.2	1	0.3	1	0.2	1	0.1
	Female	0.8 (0.5-1.1)		0.8 (0.6-1.2)		0.5 (0.2-1.3)		0.4 (0.1-1.3)	
**Charlson**	0	***1***	***0.0001***	***1***	***0.0004***	***1***	***0.004***	***1***	***0.01***
	1	***1.8 (1.1-2.8)***		***2.0 (1.3-3.2)***		***3.8 (1.0-14.1)***		***3.1 (0.8-11.3)***	
	2 or more	***2.6 (1.7-4.0)***		***2.5 (1.6-4.0)***		***7.7 (2.3-26.3)***		***5.2 (1.7-15.9)***	
**Hospital**	cont	1.1 (1.0-1.2)	0.1	1.0 (1.0-1.1)	0.3	1.0 (0.8-1.2)	0.7	1.0 (0.8-1.2)	0.7
**Stage**	I	***1***	***0.006***	***1***	***0.0002***	1	0.3	1	0.2
	II	***1.6 (0.8-3.2)***		***1.3 (0.6-2.6)***		4.4 (0.4-48.8)		5.7 (0.9-36.2)	
	III	***2.5 (1.3-5.1)***		***2.6 (1.3-5.3)***		5.8 (0.5-62.4)		3.9 (0.7-24.1)	
**Emergency**	No	***1***	***<0.001***	***1***	***<0.001***	-		-	-
	Yes	***5.0 (3.3-10.0)***		***3.3 (2.5-5.0)***					
**Resection**	Right sided	***1***	***0.01***	***1***	***0.03***	-		-	-
	Left sided	***1.4 (0.9-2.2)***		***0.7 (0.4-1.2)***					
	Sigmoid	***0.6 (0.4-1.0)***		***0.5 (0.3-0.9)***					
**Complications**	No	***1***	***<0.001***	***1***	***<0.001***	***1***	***0.001***	***1***	***0.02***
	Yes	***3.1 (2.1-4.4)***		***2.1 (1.4-3.3)***		***4.2 (1.8-9.9)***		***5.9 (1.3-26.8)***	
**Hospital stay**	Short	***1***	***<0.001***	***1***	***0.003***	***1***	***0.002***	***1***	***0.002***
	Long	***4.0 (2.7-5.9)***		***2.1 (1.4-3.2)***		***2.5 (1.0-6.3)***		***0.4 (0.1-2.1)***	
	Unknown	***0.8 (0.1-6.6)***		***0.7 (0.1-6.5)***		***9.9 (2.7-35.9)***		***8.6 (2.4-31.7)***	

For Peer Re
